# Genetic Variation May Have Promoted the Successful Colonization of the Invasive Gall Midge, *Obolodiplosis robiniae*, in China

**DOI:** 10.3389/fgene.2020.00387

**Published:** 2020-04-17

**Authors:** Yan-Xia Yao, Xing-Pu Shang, Jun Yang, Ruo-Zhu Lin, Wen-Xia Huai, Wen-Xia Zhao

**Affiliations:** Key Laboratory of Forest Protection of National Forestry and Grassland Administration/Research Institute of Forest Ecology, Environment and Protection, Chinese Academy of Forestry, Beijing, China

**Keywords:** genetic diversity, genetic differentiation, genetic structure, population colonization, invasion success

## Abstract

Invasive species often cause serious economic and ecological damage. Despite decades of extensive impacts of invasives on bio-diversity and agroforestry, the mechanisms underlying the genetic adaptation and rapid evolution of invading populations remain poorly understood. The black locust gall midge, *Obolodiplosis robiniae*, a highly invasive species that originated in North America, spread widely throughout Asia and Europe in the past decade. Here, we used 11 microsatellite DNA markers to analyze the genetic variation of 22 *O. robiniae* populations in China (the introduced region) and two additional US populations (the native region). A relatively high level of genetic diversity was detected among the introduced populations, even though they exhibited lower diversity than the native US populations. Evidence for genetic differentiation among the introduced Chinese populations was also found based on the high *Fst* value compared to the relatively low among the native US populations. Phylogenetic trees, structure graphical output, and principal coordinate analysis plots suggested that the Chinese *O. robiniae* populations (separated by up to 2,540 km) cluster into two main groups independent of geographical distance. Genetic variation has been observed to increase rapidly during adaptation to a new environment, possibly contributing to population establishment and spread. Our results provide insights into the genetic mechanisms underlying successful invasion, and identify factors that have contributed to colonization by an economically important pest species in China. In addition, the findings improve our understanding of the role that genetic structure plays during invasion by *O. robiniae*.

## Introduction

*Obolodiplosis robiniae* (Haldeman, 1847) (Diptera: Cecidomyiidae) is a North American species of gall midge that has recently been extensively introduced throughout Asia and Europe (CABI/EPPO, 2011) and is continuously expanding its range (Cierjacks et al., 2013; Stalazs, 2014; Badmin, 2016; Kostro-Ambroziak and Mieczkowska, 2017). It is specifically associated with host plants from the genus *Robinia* (Fabaceae) (Stalazs, 2014). Its main host is *Robinia pseudoacacia* although it is occasionally found on *R. pseudoacacia* cv. ‘Frisia’ (Badmin, 2016). The gall midge causes leaf rolling and premature leaf shedding, resulting in the deterioration of the host and increased susceptibility to other pests, including wood borers such as longhorn beetles (Yang et al., 2006).

*Obolodiplosis robiniae* was first recorded in China (Qinhuangdao City, Hebei Province) in 2004 (Yang et al., 2006) and has since spread extensively. Its primary host (*R. pseudoacacia*) has been planted extensively across China, and *O. robiniae* is now found in most of these areas (Shang et al., 2015a). Chinese *O. robiniae* populations may produce between four and six generations per year (Wang, 2009; Mu et al., 2010; Shao et al., 2010; Liu, 2014), which is significantly higher than the rate in regions beyond China. For example, *O. robiniae* produces three to four generations per year in Italy (Duso et al., 2005) and Serbia (Mihajlovic et al., 2008), and a maximum of three generations per year in Korea (Lee et al., 2009).

Genetic diversity and population structure are important factors affecting the colonization of invasive species (Amouroux et al., 2013; Horst and Lau, 2015; Zhao et al., 2015). Invasive species often exhibit low genetic diversity during founding events, as new habitats are typically colonized by only a few individuals, representing a small proportion of the allelic diversity present in the source population (Nei et al., 1975; Tsutsui et al., 2003). However, when the founding individuals originate from multiple source populations, the genetic diversity of the founder population can be relatively high (Davis, 2009). This can contribute to invasion success by facilitating local adaptation to new environments and increasing new trait diversity (Facon et al., 2006). Besides, the invaders can rapidly evolve in isolation from other individuals of the same species when they were introduced into the new environments (Lee, 2002; Launey et al., 2010).

DNA-based molecular markers have been extensively used to examine the genetic diversity and population structure of a wide range of species. Microsatellite DNA markers (simple sequence repeats, SSRs) are suitable for routine genetic diversity analyses (Varshney et al., 2007; Kong et al., 2014; Kim et al., 2017), as they are ubiquitous among eukaryotes (Sharma et al., 2007), co-dominantly inherited, and highly polymorphic (Zong et al., 2015). Moreover, microsatellite analysis can yield valid results and improved phylogenetic trees compared to analyses involving other molecular markers (Schemerhorn et al., 2015). Due to their feasibility and practicality, microsatellite markers have been widely used in population genetics and ecological studies of various insects (Bonizzoni et al., 2000; Mezghani-Khemakhem et al., 2012; Anjos et al., 2016; Retamal et al., 2016; Duan et al., 2017; Kim et al., 2017; Simonato et al., 2019), including several invasive gall midge species (Bentur et al., 2011; Amouroux et al., 2013).

Previously, Shang et al. (2015b) investigated the genetic variation among Chinese *O. robiniae* populations using a partial mitochondrial DNA cytochrome c oxidase subunit I (COI) sequence marker. However, only 10 individuals exhibiting haplotypic variation and a mere four haplotypes were detected in 560 *O. robiniae* samples. Thus, the genetic mechanisms behind successful invasion, the genetic structure in the process of colonization, and the phylogenetic relationships among the Chinese *O. robiniae* populations remain poorly understood.

Accordingly, to gain further insight into the genetic structure of the Chinese *O. robiniae* populations and ascertain how the species has spread widely in new regions, we used 11 microsatellite markers to analyze the genetic structure of 22 Chinese *O. robiniae* populations. Two native populations from the United States (US) were also assessed based on the same loci for comparison with the Chinese populations, in order to explain how genetic diversity is altered during the invasion process.

## Materials and Methods

### Sample Collection

**FIGURE 1:**
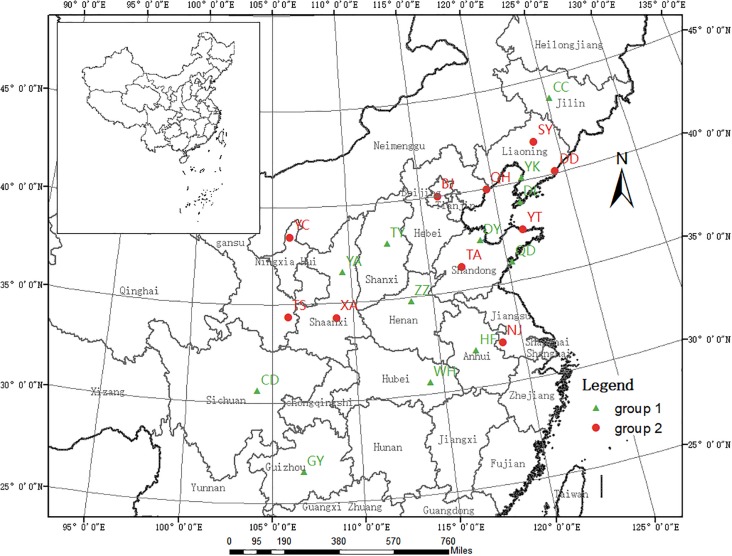
Geographic locations of the 22 Chinese *Obolodiplosis robiniae* populations sampled. Population codes are listed in Table 1. (The source map was downloaded from the website http://www.webmap.cn/commres.do?method=dataDownload).

We collected the gall midge larvae and pupae contained within rolled leaves of host trees growing in 22 cities across China (Figure 1). Generally, the rolled leaves were randomly picked from different trees; however, when infestation was low, individual trees were singled out for sample collection. Following collection, the rolled leaves were immediately transported to the laboratory in 60 cm × 40 cm plastic bags, in which they were maintained until adult emergence. From the samples collected at each location, 20 larger adults were selected, placed into a 1.5-mL centrifuge tube, and stored at −20°C for subsequent DNA extraction. Additionally, eight *O. robiniae* adults from two regions of the United States were obtained from the Quarantine Lab at the Institute of Forest Ecology, Environment, and Protection in the Chinese Academy of Forestry. Details of the sample collection and population codes are listed in Table 1.

**TABLE 1:** Location of *Obolodiplosis robiniae* populations and the sample size used in this study.

Number	Code	Site	Latitude (N)	Longitude (E)	Altitude	Sample size
(1)	BJ	Beijing	40°00.184′	116°14.363′	76	20
(2)	CC	Changchun, Jilin	43°53.851′	125°16.329′	218	20
(3)	CD	Chengdu, Sichuan	30°38.245′	104°07.334′	510	20
(4)	DD	Dandong, Liaoning	40°06.906′	124°21.536′	33	20
(5)	DL	Dalian, Liaoning	38°58.531′	121°36.800′	67	20
(6)	DY	Dongying, Shandong	37°26.366′	118°34.448′	17	20
(7)	GY	Guiyang, Guizhou	26°33.531′	106°45.003′	1090	20
(8)	HF	Hefei, Anhui	31°52.824′	117°11.639′	39	20
(9)	NJ	Nanjing, Jiangsu	32°03.426′	118°50.820′	90	20
(10)	QD	Qingdao, Shandong	36°03.367′	120°20.934′	24	20
(11)	QH	Qinhuangdao, Hebei	39°56.161′	119°35.411′	17	20
(12)	SY	Shenyang, Liaoning	41°50.438′	123°25.690′	51	20
(13)	TA	Taian, Shandong	36°12.225′	117°07.104′	208	20
(14)	TS	Tianshui, Gansu	34°21.405′	106°00.034′	1460	20
(15)	TY	Taiyuan, Shanxi	37°54.592′	112°31.811′	798	20
(16)	WH	Wuhan, Hubei	30°36.733′	114°17.772′	40	20
(17)	XA	Xian, Shaanxi	34°15.474′	108°58.938′	428	20
(18)	YA	Yanan, Shaanxi	36°35.633′	109°29.535′	1121	20
(19)	YC	Yinchuan, Ningxia	38°28.933′	106°11.983′	1115	20
(20)	YK	Yingkou, Liaoning	40°12.432′	122°04.413′	15	20
(21)	YT	Yantai, Shandong	37°32.024′	121°25.657′	9	20
(22)	ZZ	Zhengzhou, Henan	34°48.509′	113°42.266′	95	16
(23)	US_f	Finger Lakes, NY, United States	42°45′	−76°41.4′W	–	5
(24)	US_g	Goat Island, NY, United States	43°48′	−79°42′W	–	3

### DNA Extraction and Microsatellite Analyses

Genomic DNA was extracted from the entire *O. robiniae* body following the instructions described by Zhou et al. (2007) and stored at −20°C until needed. The 14 microsatellite loci (W3, W5, W8, W29, W31, W33, W35, W41, W46, W82, W83, W116, W126, and W132) developed by Yao et al. (2015) were initially selected to analyze the genotypes of 20 individuals per collection site (the exception being Zhengzhou, for which 16 individuals were analyzed) (Table 1). For each sample, we attempted to amplify all 14 loci; however, after two attempts, we were unable to amplify five loci (W29, W35, W41, W46, and W116) for many individuals; thus, these loci were not used in subsequent analyses. However, we assessed the applicability of two additional loci (W6 and W107; GenBank numbers: KP260520 and KP260530) that were not characterized by Yao et al. (2015), and we detected sufficient polymorphism among the analyzed samples. Hence, a total of 11 loci were used to genotype 444 *O. robiniae* individuals.

Microsatellite amplifications were performed in a 15 μL reaction volume containing 1 μL genomic DNA (10 ng), 1 μL of each primer (5 μmol/L), 7.5 μL 2X Taq PCR Master Mix (TIANGEN, Beijing, China), and 4.5 μL ddH_2_O. The forward primer of each primer pair was labeled with a fluorescent dye (HEX, ROX, FAM, or TMARA; Sangon Biotech, Shanghai, China). The microsatellite cycling protocol was: 5 min at 95°C (initial denaturation step); followed by 30 cycles of 94°C for 30 s, 53°C (W3, W5, W6, and W8) or 56°C (the remaining loci) for 45 s, 72°C for 45 s, extension at 72°C for 10 min, and finally maintained at 16°C. PCR products were examined using a DNA analyzer (Applied Biosystems, Waltham, CA, United States) and the results were analyzed using Genotyping was carried out using a 3730xl automated DNA sequencer (Applied Biosystems, Waltham, CA, United States). Alleles were scored using GeneMarker software version 2.2. (Softgenetics LLC, State College, PA, United States).

### Data Analyses

Genetic diversity was estimated by basic statistical analyses including number of alleles (*Na*), effective number of alleles (*Ne*), Shannon’s information index (*I*), observed heterozygosity (*Ho*), expected heterozygosity (*He*), and Nei’s (1973) expected heterozygosity (*Nei*), which were calculated using GenePop software version 4.3 (Rousset, 2008); genotype number (*GN*), gene diversity (*GD*), and polymorphism information content (*PIC*) were calculated using PowerMarker software version V3.25 (Liu and Muse, 2005). *F*-statistics and gene flow for each locus across populations were performed using PopGene software version 1.32 (Yeh et al., 2018). Deviations from the Hardy–Weinberg equilibrium (HWE) based on the Markov chain algorithm (10,000 steps) and linkage disequilibrium (LD) (10,000 permutations) were also examined by GenePop software. The genetic relationships between populations were assessed using a neighbor-joining dendrogram generated by PowerMarker and Molecular Evolutionary Genetics Analysis across Computing Platforms (MEGA X) (Kumar et al., 2018).

Populations differentiation was assessed by pairwise *Fst* values (based on 999 permutations) and gene flow (*Nm*) through AMOVA (analysis of molecular variance) approach which were performed using the Arlequin program version 3.5 (Excoffier and Lischer, 2010). The analyses can estimate variance and partitioning of the within- and among-population. Genetic structure analysis was performed with 100,000 Markov Chain Monte Carlo repetitions after a burn-in period of 200,000 interactions for each group number (*K*) using STRUCTURE software version 2.3.4 (Pritchard et al., 2000). The number of subpopulations (*K*) was assumed to be from 1 to 22, without admixture and with correlated allele frequencies. To determine the most likely number of subpopulations, the optimum *K*-value was obtained by calculating the Δ*K* value (Evanno et al., 2005).

In addition, the Mantel test was conducted using the GenALEx 6.5 program (Peakall and Smouse, 2012) to determine correlations between Nei’s genetic distance [was calculated using GenePop software based on Nei (1978)] and both geographical distance (km) and altitude (m). Significance was assessed by conducting 999 permutations. Moreover, a principal coordinate analysis (PCoA) was conducted using the same software.

## Results

### Microsatellite Polymorphism and Diversity

In this study, locus polymorphism and diversity were determined based on 22 Chinese populations using 11 microsatellite markers, with each population consisting of 20 individual samples (except Zhengzhou, with 16 samples) (Table 1). Amplifying these microsatellite markers loci led to 436 polymorphic bands (Supplementary Table S1), representing 202 genotypes, ranging from 3 to 54 per primer pair. As shown in Table 2, there were 72 alleles among the 22 populations; the number of alleles (*Na*) observed per locus varied from 2 (W83 and W107) to 14 (W3 and W5), with a mean of 6.5 per locus. The effective numbers of alleles (*Ne*) varied from 1.4087 (W83) to 7.9971 (W3), with an average of 3.7255 per locus. The gene diversity index (*GD*) per locus ranged from 0.2901 (W83) to 0.8755 (W3), with an average of 0.6511, indicating that a high level of information was provided by the 11 microsatellite markers. Shannon’s information index (*I*) ranged from 0.4767 (W83) to 2.2573 (W3), with a mean of 1.3188. The polymorphism information content (*PIC*) for the microsatellite loci ranged from 0.2494 (W83) to 0.8627 (W3), with an average of 0.6031. The observed heterozygosity (*Ho*) ranged from 0.2271 (W107) to 0.7477 (W3) and expected heterozygosity (*He*) ranged from 0.2905 (W83) to 0.8760 (W3). For each locus, both *Na* and *Ho* values markedly changed among populations, whereas, *He* value minorly altered (Supplementary Figure S1). In addition, when we evaluated the within-sample HWE deviations for each locus across all 22 Chinese populations using the Markov chain algorithm (10,000 steps), we detected significant deviation from the expected value (*p* < 0.05) in 68 of 242 tests (28.10%) (Supplementary Table S2). Examination of genotypic LD between all pairs of alleles across all 22 populations, based on a permutation procedure (10,000 permutations), revealed a significant LD (*p* < 0.05) in 251 of 1210 tests (20.74%) from 11 loci in the 22 Chinese populations.

**TABLE 2:** Polymorphism of microsatellite loci across Chinese *O. robiniae* populations.

Locus	*Na*	*GN*	*Ne*	*GD*	*I*	*Ho*	*He*	*PIC*
W3	14	54	7.9971	0.8755	2.2573	0.7477	0.8760	0.8627
W5	14	42	5.8768	0.8295	1.9743	0.7067	0.8308	0.8085
W6	4	6	2.2687	0.5601	0.9575	0.3471	0.5599	0.4973
W8	7	18	4.3609	0.7712	1.6184	0.3701	0.7716	0.7367
W31	5	14	4.2747	0.7661	1.5224	0.6697	0.7669	0.7281
W33	7	20	3.6391	0.7249	1.4660	0.6506	0.7260	0.6835
W82	5	12	3.3448	0.7010	1.3193	0.3532	0.7018	0.6487
W83	2	4	1.4087	0.2901	0.4767	0.3005	0.2905	0.2494
W107	2	3	1.6205	0.3829	0.5710	0.2271	0.3833	0.3096
W126	4	6	2.0018	0.5005	0.7223	0.4398	0.5010	0.3809
W132	8	23	4.1874	0.7603	1.6214	0.4207	0.7621	0.7284
Mean	6.5	18.3636	3.7255	0.6511	1.3188	0.4757	0.6518	0.6031

*Number of alleles (Na), genotype number (GN), effective number of alleles (Ne), gene diversity (GD), Shannon’s information index (I), observed heterozygosity (Ho), expected heterozygosity (He), polymorphism information content (PIC).*

### Population Genetic Diversity

The genetic diversity of 22 Chinese populations and two US populations was assessed. Six indices of genetic diversity (*Na*, *Ne*, *I*, *Ho*, *He*, and *Nei*) were evaluated. As shown in Table 3, for each Chinese population across all loci, the means of the above indices except *Na* were moderately or considerably lower than those of the native US populations, although the sample size of the Chinese populations (40) was markedly higher than that of the US populations (8). Regarding the three most important indices, *I*, *He*, and *Nei*, the lowest Chinese values (*I* = 0.7847, *He* = 0.4279, *Nei* = 0.4172) occurred in the DY population, and the highest (*I* = 1.1103, *He* = 0.6162, *Nei* = 0.6008) in the TS population. Increased values of these three indices occurred in the SY (*I* = 1.058, *He* = 0.6157, *Nei* = 0.6003), YT (*I* = 1.0761, *He* = 0.5825, *Nei* = 0.5677), and DD (*I* = 1.016, *He* = 0.5895, *Nei* = 0.5748) populations. The inbreeding coefficient (*Fis*) ranged from −0.0295 (TY) to 0.2011 (CD), with significant various observed for each locus among populations. Both US populations exhibited high *He* values of 0.6544 (US_f) and 0.6606 (US_g).

**TABLE 3:** Genetic diversity of the *O. robiniae* populations across 11 microsatellite loci.

Code	Sample size	*Na*	*Ne*	*I*	*Ho*	*He*	*Nei*	*Fis*
BJ	40	3.3636	2.375	0.868	0.4591	0.4928	0.4805	0.0445
CC	40	3.7273	2.5568	0.9673	0.4364	0.5425	0.529	0.1751
CD	40	3.4545	2.1684	0.8976	0.4136	0.531	0.5177	0.2011
DD	40	3.5455	2.651	1.016	0.5591	0.5895	0.5748	0.0273
DL	40	3.1818	2.3375	0.8675	0.5636	0.5026	0.49	–0.1503
DY	40	3.2727	2.2412	0.7847	0.4409	0.4279	0.4172	–0.0569
GY	40	3.5455	2.0894	0.8164	0.3864	0.4705	0.4588	0.1578
HF	40	3.1818	2.5997	0.9554	0.4909	0.5742	0.5599	0.1232
NJ	40	3.8182	2.4202	0.9633	0.4364	0.5408	0.5273	0.1724
QD	40	3.3636	2.2096	0.8265	0.3682	0.4647	0.4531	0.1874
QH	40	3.6364	2.2661	0.9192	0.5318	0.5233	0.5102	–0.0423
SY	40	3.7273	2.797	1.058	0.5455	0.6157	0.6003	0.0914
TA	40	3.3636	2.0576	0.8347	0.439	0.4792	0.4672	0.0603
TS	40	4.0909	2.8428	1.1103	0.5045	0.6162	0.6008	0.1602
TY	40	3.6364	2.3278	0.9101	0.5273	0.5253	0.5122	–0.0295
WH	40	3.7273	2.5723	0.9797	0.5256	0.5599	0.5459	0.0371
XA	40	3.5455	2.535	0.9503	0.5182	0.5492	0.5355	0.0323
YA	40	3.8182	2.7608	0.9798	0.4199	0.5355	0.5221	0.1958
YC	40	3.8182	2.4284	0.9586	0.467	0.5369	0.5234	0.1078
YK	40	4.4545	2.6524	1.0623	0.4682	0.567	0.5528	0.1531
YT	40	4.2727	2.9205	1.0761	0.5081	0.5825	0.5677	0.1049
ZZ	30	3.4545	2.3364	0.912	0.4516	0.5346	0.5168	0.1261
CN mean	40	3.6363	2.4612	0.9415	0.4755	0.5346	0.5192	–
US_f	10	4.0909	3.2336	1.1472	0.5	0.6544	0.5875	0.1489
US_g	6	3.0909	2.6363	0.9632	0.5152	0.6606	0.5505	0.0642
US mean	8	3.5909	2.935	1.0552	0.5076	0.6575	0.569	–

*Number of alleles (Na), effective number of alleles (Ne), Shannon’s information index (I), observed heterozygosity (Ho), expected homozygosity (He), Nei’s (1973) expected heterozygosity (Nei), inbreeding coefficient (Fis).*

### Genetic Differentiation in the Chinese Populations

The *Fst* per locus ranged from 0.1357 to 0.3770, with an average of 0.1994, and the *Fis* per locus ranged from −0.0037 (W3) to 0.3364 (W82) with an average of 0.0873 alleles per locus across populations (Table 4). Gene flow (*Nm*) ranged from 0.3093 at W31 to 1.5921 at W33 and averaged 1.0036. Meanwhile, the pairwise *Fst* (*p* < 0.001) values (Supplementary Table S3) between populations ranged from 0.022 (HF and WH) to 0.377 (BJ and GY), with an average value of 0.183. A total of 135 of the 213 *Fst* values (63.38%) were >0.15, while 48 (22.54%) were >0.25, which suggests that significant genetic differentiation exists among the sampling sites and there is some restriction in gene flow between them (Table 4). The most noticeable genetic differentiation occurred between BJ and GY (*Fst* = 0.377), followed by GY and QH (*Fst* = 0.372), then DY and QH, DY and GY (*Fst* = 0.361 for both). According to the coefficient of genetic differentiation (*Fst* = 0.1830, *p* < 0.001), genetic variation within populations (81.66%) was substantially higher than that among populations (18.34%) (*p* < 0.001) (Table 5). Gene flow (*Nm*) ranged from 0.414 (BJ and GY) to 11.05 (HF and WH) (Supplementary Table S4), with an average of 1.113 (Table 5). All investigated loci contributed to the population differentiation (*p* < 0.001 for each individual locus). Regarding the native US populations, they had a relatively low *Fst* value (0.085, *p* < 0.03), a high *Nm* value (2.685), and variation within populations of 91%, while variation among populations was 9% (*p* < 0.02). This further indicates the existence of extensive genetic differentiation among the introduced populations.

**TABLE 4:** Summary of *F* statistics and gene flow for each locus.

Locus	*Fis*	*Fst*	*Nm*
W3	–0.0037	0.1487	1.4307
W5	0.0026	0.1460	1.4629
W6	0.0040	0.3770	0.4130
W8	0.2929	0.3198	0.5317
W31	–0.0441	0.1603	1.3093
W33	–0.0364	0.1357	1.5921
W82	0.3364	0.2379	0.8007
W83	–0.1980	0.1367	1.5792
W107	0.3182	0.1402	1.5337
W126	–0.0165	0.1379	1.5634
W132	0.2870	0.2230	0.8745
Mean	0.0873	0.1994	1.0036

*Gene flow (Nm) estimated based on Nm = 0.25(1 – Fst)/Fst.*

**TABLE 5:** Population genetic variance revealed by 11 microsatellite loci through AMOVA analysis.

Source	Degree of freedom	Sum of squared deviations	Mean squared deviations	Variance component estimates	Percentage of variation
**Chinese populations**					
Among populations	21	622.846	29.659	0.665	18%
Among individuals	414	1371.394	3.313	0.353	10%
Within individuals	436	1136.500	2.607	2.607	72%
Total	871	3130.740		3.624	100%
*Fst*	0.183	0.001			
*Nm*	1.113				
*P*-value	<0.001				
**US populations**					
Among populations	1	7.496	7.496	0.354	9%
Among individuals	6	29.067	4.844	1.047	25%
Within individuals	8	22.000	2.750	2.750	66%
Total	15	58.563		4.151	100%
*Fst*	0.085				
*Nm*	2.685				
*P*-value	<0.03				

### Genetic Relationships and Population Structure Analysis

**FIGURE 2:**
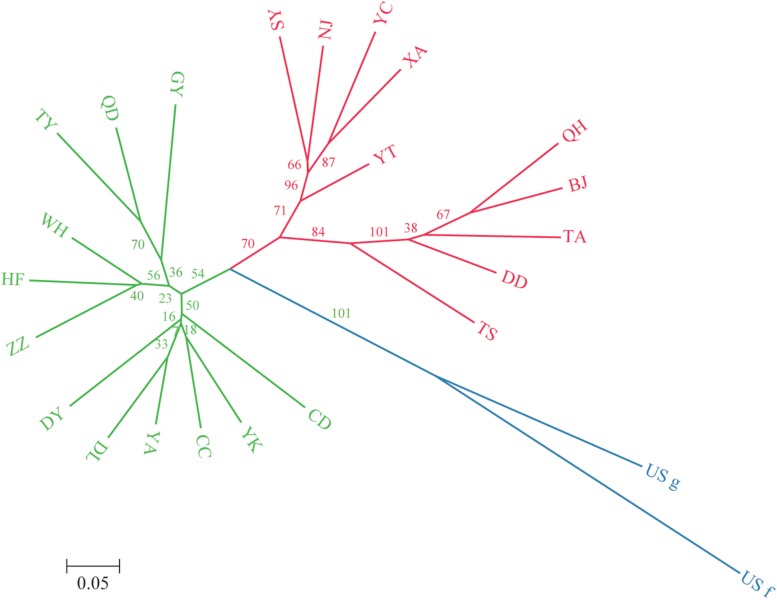
Unrooted neighbor-joining dendrogram of the 24 *O. robiniae* populations based on Nei’s distance using the allele frequencies of 11 microsatellite loci. Green represents the subpopulations of group 1, red represents the subpopulations of group 2, and blue represents the US populations.

A dendrogram depicting the genetic relationships among the 22 Chinese populations was constructed based on the microsatellite data (Figure 2). The populations were divided into two main clusters, and each cluster was further separated into several sub-clusters. Group I contained populations from Northeast China (Jilin Province) to Southwest China (Guizhou Province), including Liaoning (YK, DL), Jilin (CC), Shanxi (TY), Shaanxi (YA), Shandong (DY, QD), Henan (ZZ), Sichuan (CD), Hubei (WH), Anhui (HF), and Guizhou (GY) provinces. Group II contained populations from North China (with the exception of the NJ population), including Beijing (BJ), Liaoning (SY, DD), Hebei (QH), Shandong (YT, TA), Shaanxi (XA), Gansu (TS), Ningxia (YC), and Jiangsu (NJ) provinces. Besides, some subdivided populations were clustered according to their spatial distribution, such as GY, HF, and WH located in the south of southern China, which clustered together in group I, whereas TA, DD, BJ, and QH located around Bohai Bay clustered together in group II.

**FIGURE 3:**
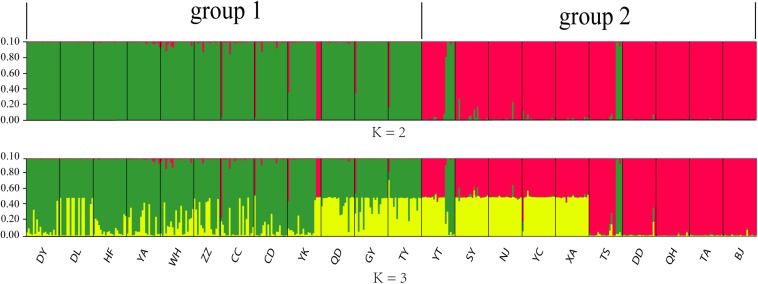
Graphical output of the STRUCTURE analysis representing hierarchical data analyses to determine the number of genetic subpopulations (*K*) of *O. robiniae*. Each individual is represented by a single vertical bar.

The 436 Chinese *O. robiniae* samples were further assessed for population stratification using STRUCTURE software. Microsatellite data were analyzed with possible cluster numbers (*K*-values) ranging from 1 to 22. Δ*K* was clearly maximized when *K* = 2 (Δ*K* = 4298.3743), indicating the occurrence of two distinct groups among the 22 populations (Figure 3), which validates the dendrogram-based grouping, and the second clade was grouped according to approximate geographical area. These results suggested different degrees of introgression in the populations, detected as differences in allelic frequencies among the populations. In addition, greater structuring (*K* = 3) revealed that QD, GY, and TY in group I had certain structural similarities to YT, SY, NJ, YC, and XA in group II.

**FIGURE 4:**
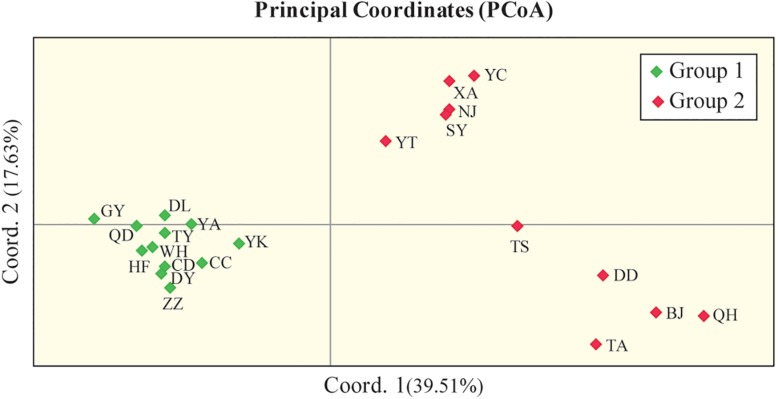
Principal coordinate analysis (PCoA) of 436 Chinese *O. robiniae* individuals showing two distinct clusters of populations. Each population is represented by a diamond. Coord.1 (39.51%) and Coord.2 (17.63%) refer to the first and second principal component, respectively.

In addition, PCoA based on the marker genotypes also revealed two distinct clusters of the Chinese populations (Figure 4), which were partly related to their geographical regions (group II contained populations from North China). The Mantel test revealed non-significant negative correlations between Nei’s genetic distance (Supplementary Table S5) and geographical distance (km) (*r* = −0.02, *P* = 0.456; Supplementary Figure S2), and between Nei’s genetic distance and altitude (*r* = −0.026, *P* = 0.419; Supplementary Figure S3), which indicates that genetic differentiation in the 22 Chinese populations may not be caused by geographical isolation.

## Discussion

In the present study, we detected a high degree of polymorphism among the assessed microsatellite loci. We also identified a relatively high level of genetic diversity among Chinese *O. robiniae* populations across all loci, with the average expected heterozygosity (He) and Nei’s (1973) expected heterozygosity (*Nei*) being 0.5346 and 0.5192, respectively. The highest *He* was 0.6606, which occurred in the US_g population despite the fact that it consisted of only three *O. robiniae* individuals. Nonetheless, *He*, *Nei* (Shang et al., 2016), gene diversity index (*GD*), and polymorphism information content (*PIC*) (Ren et al., 2014) are minimally influenced by sample size. Our results indicate significant differences in genetic diversity within the Chinese populations, as well as between the native and invasive populations. Shang et al. (2015b) detected a relatively low level of genetic diversity among Chinese *O. robiniae* populations using a COI marker. This discrepancy suggests that COI markers may be less suitable than microsatellite DNA markers for population analyses of a new invasive species, such as Chinese *O. robiniae*, as is the case with another invasive cecidomyiid, *Procontarinia mangiferae* (Amouroux et al., 2013).

The relatively high level of genetic diversity has likely contributed to the spread of *O. robiniae* across China in the past decade to the extent that this species is now established in most regions where its host exists (Shang et al., 2015a). *O. robiniae* has a short history in China, with initial detection occurring in 2004 (Yang et al., 2006), whereas its host was introduced over a century ago (Xu and Yang, 2006). Moreover, for the 22 Chinese populations analyzed, our results show a relatively high level of genetic differentiation (*Fst* = 0.1830) among populations from sites separated by distances of up to 2,540 km. Hence, the relatively high genetic diversity likely caused by significant differentiation among populations has been conducive to the rapid colonization and establishment of *O. robiniae* in China, and it is an important factor contributing to the successful invasion of *O. robiniae*.

The observed population differentiation likely resulted from rapid evolution during adaptation to the new environment. Invasive species may evolve rapidly in response to selection pressures driven by novel habitats (Sakai et al., 2001; Lee, 2002; Ochocki and Miller, 2017), and such rapid genetic adaptation might be important for invasive species (Shine et al., 2011) in order to increase fitness and invasion success (Suarez and Tsutsui, 2008; Roux and Wieczorek, 2009). Increasing the success of both their initial establishment and subsequent range expansion is a particularly effective strategy for introduced populations, as was shown for several invasive species during their colonization processes (Simberloff et al., 2013; Ochocki and Miller, 2017; Wu et al., 2019). Hence, differentiation among Chinese populations was likely accelerated by the rapid evolution of adaptations to the new environments, promoting successful invasion by *O. robiniae*.

Furthermore, high levels of genetic diversity in an invasive species might be caused by multiple introductions or large founding populations. It is thought that multiple introductions are associated with increased diversity because they supply increased variation and new genetic communities (Dlugosch and Parker, 2008). Multiple introductions are considered to produce invasive populations that are much more genetically diverse than a single source population (Sakai et al., 2001). As such, the successful establishment and invasion of many invasive species have been attributed to multiple introductions (Meixner et al., 2002; Kolbe et al., 2004; Cheng et al., 2008; Zalewski et al., 2010; Michaelides et al., 2018). For *O. robiniae*, in light of the short history in China, its high genetic diversity and colonization success might be also related to multiple independent invasive events.

Many studies have shown that the genetic structures of invasive species are well developed in their new ranges (Zeisset and Beebee, 2003; Herborg et al., 2007; Rollins et al., 2009; Zalewski et al., 2010). Indeed, the genetic diversity of invasive species is often higher than that of native populations (Marrs et al., 2008). In light of this, our neighbor-joining dendrogram and Bayesian STRUCTURE (*K* = 2) analyses indicated that the Chinese *O. robiniae* populations are divided into two independent clusters, although this division appears to be unrelated to geographical distribution. Meanwhile, gene flow was found among some Chinese populations, with QD, GY, and TY in group I having highly similar structures to YT, SY, NJ, YC, and XA in group II. In addition, some subgroups (GY, HF, and WH; TA, DD, BJ, and QH) were clustered according to their geographical distribution, which likely represents different routes of spread in the new environment. Furthermore, our results revealed that each group of the Chinese *O. robiniae* populations exhibited differences in geographical distribution and genetic distance, suggesting that the two groups do in fact represent two different sources. This implies the introduced populations likely experienced two independent invasive events, which initially shaped the genetic structure of the Chinese *O. robiniae* populations.

On the other hand, the fact that the division of the two groups was unrelated to geographical distribution (particularly for group I, which contained numerous genetically similar populations located in different geographical regions) suggests that human activity is likely another important contributing factor. Transport, business trips, and long-distance vacations have recently increased not only in frequency but also in distance. High levels of human-mediated dispersion can increase the genetic diversity of an invasive population, thereby substantially modifying the genetic structure and potential management units (Perkins et al., 2013); these factors decrease the success of control measures for *O. robiniae* populations.

## Data Availability Statement

All datasets generated for this study are included in the article/Supplementary Material.

## Author Contributions

Y-XY and W-XZ: conceptualization, funding acquisition, project administration, and supervision. R-ZL, W-XH, and JY: data curation. Y-XY, X-PS, and JY: formal analysis, investigation, and resources. Y-XY and X-PS: methodology. Y-XY and R-ZL: software. Y-XY, R-ZL, and W-XH: validation. Y-XY, X-PS, R-ZL, and W-XH: visualization. Y-XY, X-PS, R-ZL, W-XH, JY, and W-XZ: writing – original draft and review and editing.

## Conflict of Interest

The authors declare that the research was conducted in the absence of any commercial or financial relationships that could be construed as a potential conflict of interest.
