# Supplementary figures and images for: Genetic Variation May Have Promoted the Successful Colonization of the Invasive Gall Midge, Obolodiplosis robiniae, in China
Source: Front Genet. 2020 Apr 17;11:387. doi: 10.3389/fgene.2020.00387 (PMC7180195; doi:10.3389/fgene.2020.00387)

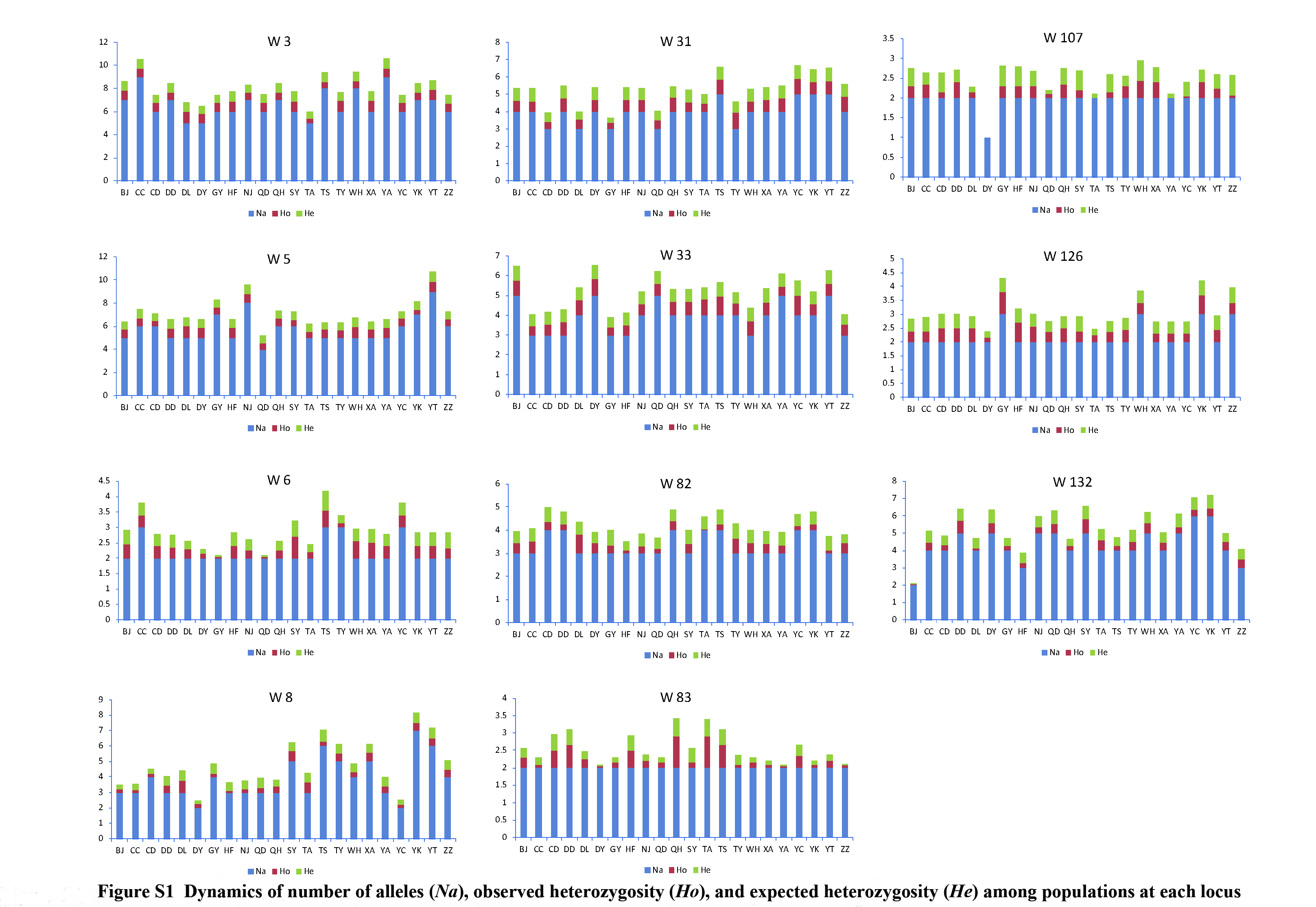

Supplement: Supplementary file 1 [file Image_1.TIF]

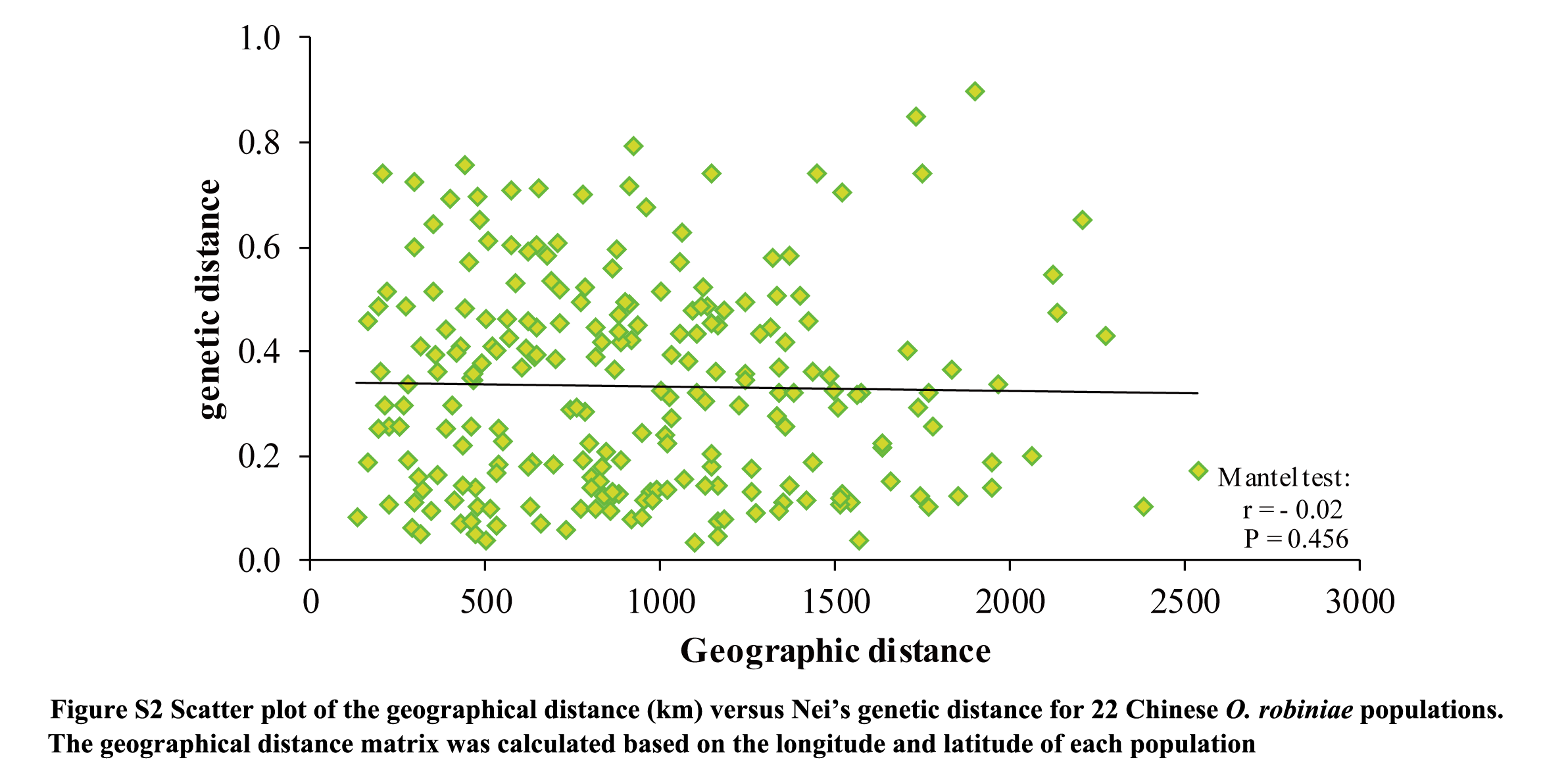

Supplement: Supplementary file 2 [file Image_2.TIF]

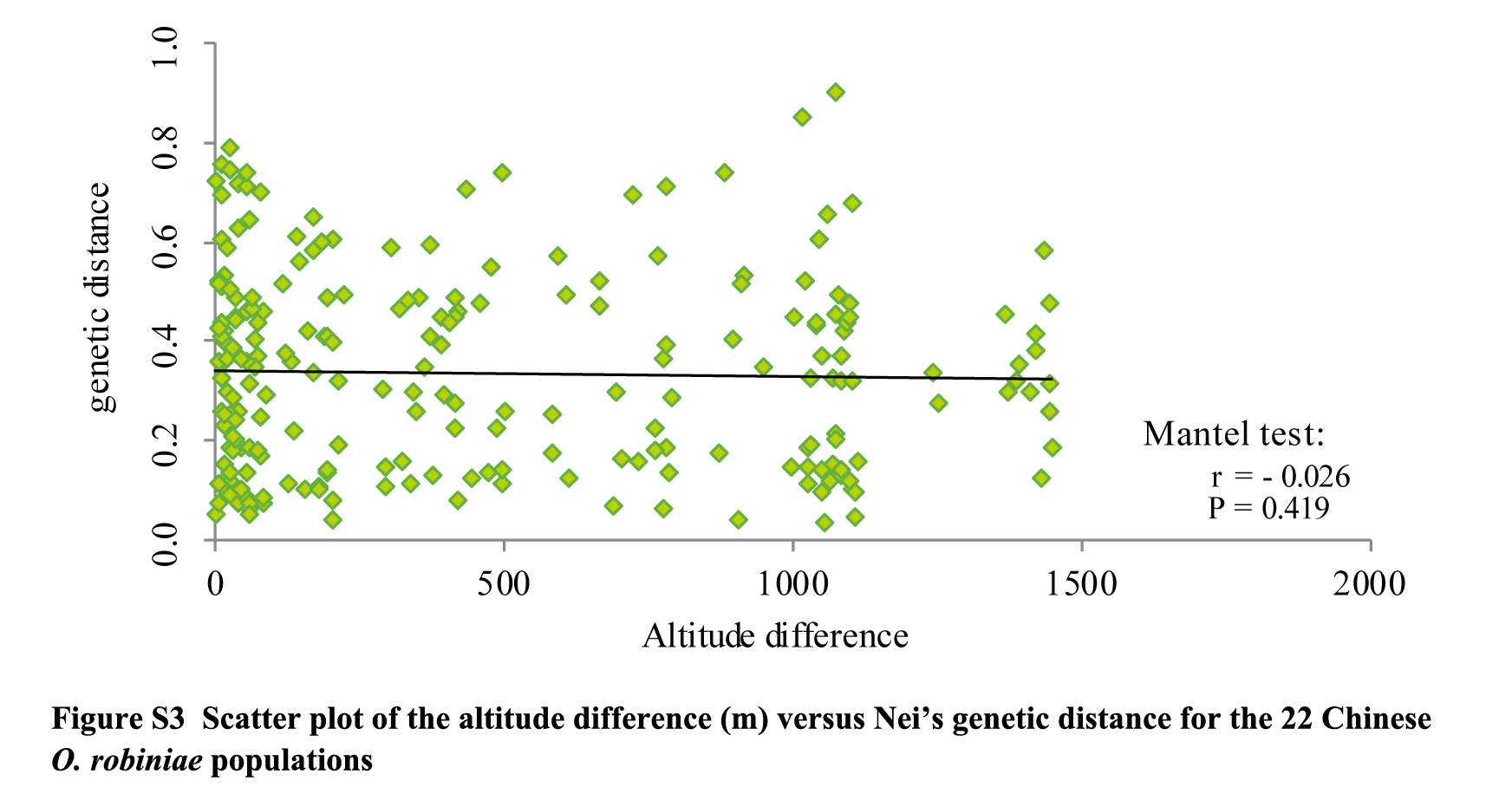

Supplement: Supplementary file 3 [file Image_3.TIF]
