# Supplementary material for: Genetic Variation May Have Promoted the Successful Colonization of the Invasive Gall Midge, Obolodiplosis robiniae, in China
Source: Front Genet. 2020 Apr 17;11:387. doi: 10.3389/fgene.2020.00387 (PMC7180195; doi:10.3389/fgene.2020.00387)
Supplement: Supplementary file 4 [file Table_1.DOC]

Table S1. Genetic diversity per locus in each of Chinese *O. robiniae* population

| **Locus** | **P.** | **BJ** | **CC** | **CD** | **DD** | **DL** | **DY** | **GY** | **HF** | **NJ** | **QD** | **QH** | **SY** | **TA** | **TS** | **TY** | **WH** | **XA** | **YA** | **YC** | **YK** | **YT** | **ZZ** |
| --- | --- | --- | --- | --- | --- | --- | --- | --- | --- | --- | --- | --- | --- | --- | --- | --- | --- | --- | --- | --- | --- | --- | --- |
| W3 | *Na* | 7 | 9 | 6 | 7 | 5 | 5 | 6 | 6 | 7 | 6 | 7 | 6 | 5 | 8 | 6 | 8 | 6 | 9 | 6 | 7 | 7 | 6 |
|  | *H*_O_*H*e | 0.800/0.814 | 0.700/0.810 | 0.750/0.668 | 0.650/0.759 | 1.000/0.780 | 0.800/0.699 | 0.750/0.675 | 0.900/0.834 | 0.600/0.693 | 0.750/0.729 | 0.650/0.791 | 0.900/0.833 | 0.400/0.579 | 0.550/0.844 | 0.950/0.741 | 0.631/0.796 | 0.950/0.819 | 0.750/0.866 | 0.736/0.671 | 0.650/0.784 | 0.894/0.815 | 0.666/0.790 |
|  | *F*_IS_ | -0.007 | 0.113 | -0.151 | 0.121 | -0.313 | -0.174 | -0.138 | -0.106 | 0.112 | -0.054 | 0.157 | -0.107 | 0.292 | 0.332 | -0.314 | 0.185 | -0.189 | 0.112 | -0.127 | 0.150 | -0.127 | 0.127 |
| W5 | *Na* | 5 | 6 | 6 | 5 | 5 | 5 | 7 | 5 | 8 | 4 | 6 | 6 | 5 | 5 | 5 | 5 | 5 | 5 | 6 | 7 | 9 | 6 |
|  | *H*_O_*H*e | 0.700/0.738 | 0.700/0.782 | 0.450/0.615 | 0.800/0.788 | 1.000/0.773 | 0.850/0.791 | 0.650/0.682 | 0.850/0.760 | 0.800/0.810 | 0.500/0.664 | 0.650/0.688 | 0.550/0.717 | 0.550/0.650 | 0.700/0.632 | 0.650/0.707 | 0.950/0.803 | 0.700/0.724 | 0.850/0.755 | 0.650/0.652 | 0.450/0.742 | 0.894/0.854 | 0.642/0.650 |
|  | *F*_IS_ | 0.027 | 0.082 | 0.250 | -0.041 | -0.326 | -0.102 | 0.022 | -0.146 | -0.012 | 0.227 | 0.031 | 0.214 | 0.132 | -0.135 | 0.058 | -0.212 | 0.008 | -0.154 | -0.021 | 0.378 | -0.074 | -0.024 |
| W6 | *Na* | 2 | 3 | 2 | 2 | 2 | 2 | 2 | 2 | 2 | 2 | 2 | 2 | 2 | 3 | 3 | 2 | 2 | 2 | 3 | 2 | 2 | 2 |
|  | *H*_O_*H*e | 0.450/0.450 | 0.400/0.405 | 0.400/0.384 | 0.350/0.409 | 0.300/0.262 | 0.150/0.142 | 0.050/0.050 | 0.400/0.431 | 0.250/0.357 | 0.050/0.050 | 0.250/0.296 | 0.700/0.507 | 0.200/0.261 | 0.550/0.626 | 0.150/0.232 | 0.550/0.409 | 0.500/0.431 | 0.400/0.384 | 0.400/0.405 | 0.400/0.431 | 0.400/0.430 | 0.333/0.508 |
|  | *F*_IS_ | -0.025 | -0.012 | -0.066 | 0.122 | -0.176 | -0.081 | -0.025 | 0.047 | 0.283 | -0.025 | 0.134 | -0.414 | 0.215 | 0.100 | 0.337 | -0.379 | -0.190 | -0.066 | -0.012 | 0.047 | 0.047 | 0.321 |
| W8 | *Na* | 3 | 3 | 4 | 3 | 3 | 2 | 4 | 3 | 3 | 3 | 3 | 5 | 3 | 6 | 5 | 4 | 5 | 3 | 2 | 7 | 6 | 4 |
|  | *H*_O_*H*e | 0.200/0.273 | 0.150/0.383 | 0.200/0.316 | 0.450/0.600 | 0.750/0.672 | 0.250/0.224 | 0.200/0.632 | 0.100/0.553 | 0.200/0.552 | 0.300/0.626 | 0.400/0.405 | 0.650/0.587 | 0.650/0.588 | 0.300/0.729 | 0.500/0.610 | 0.300/0.538 | 0.550/0.584 | 0.368/0.607 | 0.200/0.328 | 0.500/0.661 | 0.500/0.712 | 0.437/0.647 |
|  | *F*_IS_ | 0.248 | 0.598 | 0.352 | 0.231 | -0.145 | -0.142 | 0.675 | 0.814 | 0.628 | 0.509 | -0.012 | -0.135 | -0.132 | 0.578 | 0.159 | 0.428 | 0.035 | 0.377 | 0.375 | 0.224 | 0.280 | 0.302 |
| W31 | *Na* | 4 | 4 | 3 | 4 | 3 | 4 | 3 | 4 | 4 | 3 | 4 | 4 | 4 | 5 | 3 | 4 | 4 | 4 | 5 | 5 | 5 | 4 |
|  | *H*_O_*H*e | 0.650/0.685 | 0.600/0.742 | 0.400/0.548 | 0.750/0.742 | 0.550/0.447 | 0.700/0.696 | 0.350/0.304 | 0.700/0.723 | 0.700/0.667 | 0.500/0.526 | 0.800/0.655 | 0.550/0.711 | 0.450/0.544 | 0.850/0.728 | 0.950/0.644 | 0.600/0.688 | 0.700/0.701 | 0.750/0.725 | 0.900/0.770 | 0.700/0.760 | 0.750/0.791 | 0.875/0.711 |
|  | *F*_IS_ | 0.028 | 0.171 | 0.252 | -0.036 | -0.261 | -0.031 | -0.181 | 0.007 | -0.074 | 0.026 | -0.252 | 0.207 | 0.152 | -0.197 | -0.510 | 0.106 | -0.023 | -0.060 | -0.198 | 0.055 | 0.027 | -0.269 |
| W33 | *Na* | 5 | 3 | 3 | 3 | 4 | 5 | 3 | 3 | 4 | 5 | 4 | 4 | 4 | 4 | 4 | 3 | 4 | 5 | 4 | 4 | 5 | 3 |
|  | *H*_O_*H*e | 0.750/0.741 | 0.450/0.591 | 0.550/0.627 | 0.650/0.655 | 0.750/0.658 | 0.850/0.681 | 0.400/0.498 | 0.500/0.621 | 0.550/0.645 | 0.600/0.625 | 0.700/0.634 | 0.700/0.626 | 0.800/0.616 | 0.950/0.729 | 0.600/0.580 | 0.700/0.670 | 0.650/0.714 | 0.450/0.641 | 1.000/0.757 | 0.550/0.637 | 0.600/0.679 | 0.533/0.508 |
|  | *F*_IS_ | -0.038 | 0.219 | 0.100 | -0.017 | -0.169 | -0.281 | 0.177 | 0.173 | 0.125 | 0.016 | -0.131 | -0.145 | -0.330 | -0.335 | -0.059 | -0.070 | 0.066 | 0.280 | -0.353 | 0.114 | 0.094 | -0.086 |
| W82 | *Na* | 3 | 3 | 4 | 4 | 3 | 3 | 3 | 3 | 3 | 3 | 4 | 3 | 4 | 4 | 3 | 3 | 3 | 3 | 4 | 4 | 3 | 3 |
|  | *H*_O_*H*e | 0.450/0.509 | 0.500/0.579 | 0.350/0.654 | 0.250/0.542 | 0.800/0.563 | 0.450/0.472 | 0.350/0.683 | 0.150/0.367 | 0.300/0.550 | 0.200/0.471 | 0.400/0.491 | 0.400/0.614 | 0.050/0.542 | 0.250/0.657 | 0.650/0.626 | 0.450/0.562 | 0.400/0.559 | 0.350/0.573 | 0.200/0.506 | 0.250/0.547 | 0.150/0.588 | 0.437/0.377 |
|  | *F*_IS_ | 0.093 | 0.115 | 0.451 | 0.527 | -0.457 | -0.280 | 0.474 | 0.581 | 0.440 | 0.565 | 0.164 | 0.331 | 0.905 | 0.610 | -0.063 | 0.180 | 0.266 | 0.373 | 0.594 | 0.531 | 0.738 | -0.197 |
| W83 | *Na* | 2 | 2 | 2 | 2 | 2 | 2 | 2 | 2 | 2 | 2 | 2 | 2 | 2 | 2 | 2 | 2 | 2 | 2 | 2 | 2 | 2 | 2 |
|  | *H*_O_*H*e | 0.300/0.261 | 0.100/0.184 | 0.500/0.467 | 0.650/0.450 | 0.250/0.224 | 0.050/0.050 | 0.150/0.142 | 0.500/0.431 | 0.200/0.184 | 0.150/0.142 | 0.900/0.512 | 0.150/0.409 | 0.900/0.507 | 0.650/0.450 | 0.100/0.271 | 0.150/0.142 | 0.100/0.097 | 0.050/0.050 | 0.350/0.296 | 0.100/0.097 | 0.200/0.184 | 0.062/0.062 |
|  | *F*_IS_ | -0.176 | 0.444 | -0.098 | -0.481 | -0.142 | -0.025 | -0.081 | -0.191 | -0.111 | -0.081 | -0.800 | 0.623 | -0.818 | -0.481 | 0.622 | -0.081 | -0.052 | -0.025 | -0.212 | -0.052 | -0.111 | -0.032 |
| W107 | *Na* | 2 | 2 | 2 | 2 | 2 | 1 | 2 | 2 | 2 | 2 | 2 | 2 | 2 | 2 | 2 | 2 | 2 | 2 | 2 | 2 | 2 | 2 |
|  | *H*_O_*H*e | 0.300/0.466 | 0.350/0.296 | 0.150/0.481 | 0.400/0.328 | 0.150/0.142 | 0.000/0.000 | 0.300/0.513 | 0.300/0.507 | 0.300/0.384 | 0.100/0.097 | 0.350/0.409 | 0.200/0.492 | 0.000/0.097 | 0.150/0.450 | 0.300/0.261 | 0.450/0.501 | 0.400/0.384 | 0.000/0.097 | 0.050/0.357 | 0.400/0.328 | 0.250/0.357 | 0.062/0.514 |
|  | *F*_IS_ | 0.341 | -0.212 | 0.680 | -0.250 | -0.081 | - - | 0.400 | 0.393 | 0.200 | -0.052 | 0.122 | 0.583 | 1.000 | 0.658 | -0.176 | 0.079 | -0.066 | 1.000 | 0.856 | -0.250 | 0.283 | 0.874 |
| W126 | *Na* | 2 | 2 | 2 | 2 | 2 | 2 | 3 | 2 | 2 | 2 | 2 | 2 | 2 | 2 | 2 | 3 | 2 | 2 | 2 | 3 | 2 | 3 |
|  | *H*_O_*H*e | 0.400/0.431 | 0.400/0.507 | 0.500/0.508 | 0.500/0.508 | 0.500/0.431 | 0.150/0.224 | 0.800/0.512 | 0.700/0.507 | 0.550/0.450 | 0.350/0.409 | 0.500/0.430 | 0.400/0.512 | 0.250/0.224 | 0.350/0.409 | 0.450/0.409 | 0.400/0.444 | 0.300/0.430 | 0.300/0.430 | 0.300/0.430 | 0.700/0.511 | 0.450/0.501 | 0.416/0.539 |
|  | *F*_IS_ | 0.047 | 0.191 | -0.010 | -0.010 | -0.190 | 0.314 | -0.604 | -0.414 | -0.253 | 0.122 | -0.191 | 0.200 | -0.142 | 0.122 | -0.128 | 0.077 | 0.285 | 0.285 | 0.285 | -0.403 | 0.079 | 0.194 |
| W132 | *Na* | 2 | 4 | 4 | 5 | 4 | 5 | 4 | 3 | 5 | 5 | 4 | 5 | 4 | 4 | 4 | 5 | 4 | 5 | 6 | 6 | 4 | 3 |
|  | *H*_O_*H*e | 0.050/0.050 | 0.450/0.685 | 0.300/0.572 | 0.700/0.706 | 0.150/0.575 | 0.600/0.727 | 0.250/0.483 | 0.300/0.579 | 0.350/0.652 | 0.550/0.767 | 0.250/0.442 | 0.800/0.760 | 0.578/0.658 | 0.250/0.520 | 0.500/0.692 | 0.600/0.601 | 0.450/0.594 | 0.350/0.759 | 0.350/0.729 | 0.450/0.735 | 0.500/0.491 | 0.500/0.570 |
|  | *F*_IS_ | -0.025 | 0.327 | 0.462 | -0.022 | 0.732 | 0.153 | 0.469 | 0.469 | 0.449 | 0.265 | 0.420 | -0.079 | 0.097 | 0.507 | 0.259 | -0.023 | 0.224 | 0.527 | 0.507 | 0.372 | -0.044 | 0.095 |

P. means Parameter. Number of allele (*Na*), observed heterozygosity (*H*o), expected heterozygosity (*H*e) and inbreeding coefficients (*F*_IS_) in each locus.
