# Supplementary material for: Genetic Variation May Have Promoted the Successful Colonization of the Invasive Gall Midge, Obolodiplosis robiniae, in China
Source: Front Genet. 2020 Apr 17;11:387. doi: 10.3389/fgene.2020.00387 (PMC7180195; doi:10.3389/fgene.2020.00387)
Supplement: Supplementary file 5 [file Table_2.DOCX]

| **Locus** |  | **HWE for loci in each population** | | | | | | | | | | | | | | | | | | | | |
| --- | --- | --- | --- | --- | --- | --- | --- | --- | --- | --- | --- | --- | --- | --- | --- | --- | --- | --- | --- | --- | --- | --- |
|  | BJ | CC | CD | DD | DL | DY | GY | HF | NJ | QD | QH | SY | TA | TS | TY | WH | XA | YA | YC | YK | YT | ZZ |
| W3 | 0.336 | 0.184 | 0.466 | 0.010 | 1.000 | 0.729 | 0.093 | 0.686 | 0.295 | 0.678 | 0.149 | 0.817 | 0.208 | 0.001 | 0.999 | 0.066 | 0.954 | 0.252 | 0.817 | 0.030 | 0.742 | 0.129 |
| W5 | 0.340 | 0.011 | 0.003 | 0.712 | 1.000 | 0.835 | 0.470 | 0.522 | 0.003 | 0.168 | 0.365 | 0.012 | 0.305 | 0.522 | 0.344 | 0.953 | 0.611 | 0.852 | 0.049 | 0.005 | 0.199 | 0.228 |
| W6 | 0.690 | 0.025 | 0.776 | 0.442 | 1.000 | 1.000 | 0.000 | 0.569 | 0.213 | 0.000 | 0.467 | 0.988 | 0.353 | 0.063 | 0.025 | 1.000 | 0.907 | 0.776 | 0.025 | 0.569 | 0.569 | 0.193 |
| W8 | 0.147 | 0.001 | 0.012 | 0.027 | 0.784 | 1.000 | 0.000 | 0.001 | 0.001 | 0.004 | 0.614 | 0.697 | 0.051 | 0.001 | 0.089 | 0.021 | 0.481 | 0.018 | 0.129 | 0.006 | 0.051 | 0.101 |
| W31 | 0.309 | 0.124 | 0.112 | 0.324 | 1.000 | 0.462 | 1.000 | 0.448 | 0.707 | 0.058 | 0.946 | 0.082 | 0.539 | 0.928 | 1.000 | 0.208 | 0.118 | 0.547 | 0.941 | 0.015 | 0.262 | 0.911 |
| W33 | 0.702 | 0.219 | 0.118 | 0.646 | 0.930 | 0.950 | 0.216 | 0.234 | 0.069 | 0.283 | 0.777 | 0.849 | 0.981 | 0.998 | 0.022 | 0.690 | 0.154 | 0.044 | 1.000 | 0.074 | 0.178 | 0.633 |
| W82 | 0.049 | 0.253 | 0.004 | 0.001 | 1.000 | 0.657 | 0.002 | 0.008 | 0.003 | 0.001 | 0.075 | 0.025 | 0.000 | 0.000 | 0.658 | 0.292 | 0.009 | 0.011 | 0.001 | 0.001 | 0.000 | 1.000 |
| W83 | 1.000 | 0.151 | 0.794 | 1.000 | 1.000 | 0.000 | 1.000 | 0.907 | 1.000 | 1.000 | 1.000 | 0.009 | 1.000 | 1.000 | 0.151 | 1.000 | 1.000 | 0.000 | 1.000 | 1.000 | 1.000 | 0.000 |
| W107 | 0.122 | 1.000 | 0.002 | 1.000 | 1.000 | 0.000 | 0.069 | 0.073 | 0.320 | 1.000 | 0.442 | 0.010 | 0.025 | 0.004 | 1.000 | 0.493 | 0.776 | 0.025 | 0.001 | 1.000 | 0.213 | 0.001 |
| W126 | 0.569 | 0.299 | 0.649 | 0.649 | 0.907 | 0.246 | 1.000 | 0.988 | 0.947 | 0.442 | 0.907 | 0.285 | 1.000 | 0.442 | 0.851 | 0.442 | 0.188 | 0.188 | 0.188 | 0.987 | 0.493 | 0.032 |
| W132 | 0.000 | 0.002 | 0.059 | 0.235 | 0.019 | 0.132 | 0.006 | 0.001 | 0.009 | 0.029 | 0.038 | 0.611 | 0.153 | 0.001 | 0.053 | 0.569 | 0.049 | 0.000 | 0.001 | 0.001 | 0.633 | 0.139 |

Table S2. Hardy–Weinberg equilibrium (HWE) test (p-values) for each locus
