# Supplementary material for: Genetic Variation May Have Promoted the Successful Colonization of the Invasive Gall Midge, Obolodiplosis robiniae, in China
Source: Front Genet. 2020 Apr 17;11:387. doi: 10.3389/fgene.2020.00387 (PMC7180195; doi:10.3389/fgene.2020.00387)
Supplement: Supplementary file 6 [file Table_3.DOCX]

| Table S3. Pairwise *Fst* values between the 22 Chinese *O. robiniae* populations | | | | | | | | | | | | | | | | | | | | | | | | |
| --- | --- | --- | --- | --- | --- | --- | --- | --- | --- | --- | --- | --- | --- | --- | --- | --- | --- | --- | --- | --- | --- | --- | --- | --- |
|  | BJ | CC | CD | DD | DL | DY | GY | HF | NJ | QD | QH | SY | TA | TS | TY | WH | XA | YA | YC | YK | YT | ZZ |  |  |
| BJ | 0.000 | 0.001 | 0.001 | 0.001 | 0.001 | 0.001 | 0.001 | 0.001 | 0.001 | 0.001 | 0.001 | 0.001 | 0.001 | 0.001 | 0.001 | 0.001 | 0.001 | 0.001 | 0.001 | 0.001 | 0.001 | 0.001 |  |  |
| CC | 0.279 | 0.000 | 0.001 | 0.001 | 0.001 | 0.001 | 0.001 | 0.001 | 0.001 | 0.001 | 0.001 | 0.001 | 0.001 | 0.001 | 0.001 | 0.001 | 0.001 | 0.044 | 0.001 | 0.057 | 0.001 | 0.001 |  |  |
| CD | 0.320 | 0.064 | 0.000 | 0.001 | 0.001 | 0.001 | 0.001 | 0.001 | 0.001 | 0.001 | 0.001 | 0.001 | 0.001 | 0.001 | 0.001 | 0.001 | 0.001 | 0.001 | 0.001 | 0.001 | 0.001 | 0.001 |  |  |
| DD | 0.117 | 0.198 | 0.242 | 0.000 | 0.001 | 0.001 | 0.001 | 0.001 | 0.001 | 0.001 | 0.002 | 0.001 | 0.001 | 0.001 | 0.001 | 0.001 | 0.001 | 0.001 | 0.001 | 0.001 | 0.001 | 0.001 |  |  |
| DL | 0.334 | 0.071 | 0.086 | 0.240 | 0.000 | 0.001 | 0.001 | 0.001 | 0.001 | 0.001 | 0.001 | 0.001 | 0.001 | 0.001 | 0.001 | 0.001 | 0.001 | 0.052 | 0.001 | 0.002 | 0.001 | 0.001 |  |  |
| DY | 0.354 | 0.067 | 0.093 | 0.284 | 0.122 | 0.000 | 0.001 | 0.001 | 0.001 | 0.001 | 0.001 | 0.001 | 0.001 | 0.001 | 0.001 | 0.001 | 0.001 | 0.001 | 0.001 | 0.001 | 0.001 | 0.001 |  |  |
| GY | 0.377 | 0.123 | 0.172 | 0.295 | 0.144 | 0.183 | 0.000 | 0.001 | 0.001 | 0.001 | 0.001 | 0.001 | 0.001 | 0.001 | 0.001 | 0.001 | 0.001 | 0.001 | 0.001 | 0.001 | 0.001 | 0.001 |  |  |
| HF | 0.305 | 0.060 | 0.078 | 0.220 | 0.123 | 0.141 | 0.098 | 0.000 | 0.001 | 0.001 | 0.001 | 0.001 | 0.001 | 0.001 | 0.001 | 0.019 | 0.001 | 0.001 | 0.001 | 0.001 | 0.001 | 0.001 |  |  |
| NJ | 0.236 | 0.194 | 0.233 | 0.163 | 0.223 | 0.242 | 0.253 | 0.217 | 0.000 | 0.001 | 0.001 | 0.002 | 0.001 | 0.001 | 0.001 | 0.001 | 0.001 | 0.001 | 0.001 | 0.001 | 0.002 | 0.001 |  |  |
| QD | 0.351 | 0.097 | 0.158 | 0.286 | 0.078 | 0.101 | 0.126 | 0.151 | 0.216 | 0.000 | 0.001 | 0.001 | 0.001 | 0.001 | 0.002 | 0.001 | 0.001 | 0.001 | 0.001 | 0.001 | 0.001 | 0.001 |  |  |
| QH | 0.044 | 0.278 | 0.314 | 0.071 | 0.330 | 0.361 | 0.372 | 0.306 | 0.231 | 0.348 | 0.000 | 0.001 | 0.001 | 0.001 | 0.001 | 0.001 | 0.001 | 0.001 | 0.001 | 0.001 | 0.001 | 0.001 |  |  |
| SY | 0.201 | 0.164 | 0.213 | 0.135 | 0.202 | 0.249 | 0.223 | 0.161 | 0.037 | 0.209 | 0.209 | 0.000 | 0.001 | 0.001 | 0.001 | 0.001 | 0.001 | 0.001 | 0.001 | 0.001 | 0.002 | 0.001 |  |  |
| TA | 0.161 | 0.244 | 0.295 | 0.068 | 0.318 | 0.311 | 0.361 | 0.295 | 0.223 | 0.328 | 0.104 | 0.216 | 0.000 | 0.001 | 0.001 | 0.001 | 0.001 | 0.001 | 0.001 | 0.001 | 0.001 | 0.001 |  |  |
| TS | 0.175 | 0.162 | 0.170 | 0.058 | 0.186 | 0.257 | 0.270 | 0.168 | 0.148 | 0.268 | 0.139 | 0.126 | 0.160 | 0.000 | 0.001 | 0.001 | 0.001 | 0.001 | 0.001 | 0.001 | 0.001 | 0.001 |  |  |
| TY | 0.322 | 0.113 | 0.181 | 0.253 | 0.115 | 0.152 | 0.109 | 0.133 | 0.203 | 0.043 | 0.313 | 0.192 | 0.295 | 0.228 | 0.000 | 0.001 | 0.001 | 0.001 | 0.001 | 0.001 | 0.001 | 0.001 |  |  |
| WH | 0.290 | 0.059 | 0.083 | 0.221 | 0.119 | 0.099 | 0.069 | 0.022 | 0.187 | 0.109 | 0.303 | 0.155 | 0.285 | 0.184 | 0.112 | 0.000 | 0.001 | 0.001 | 0.001 | 0.001 | 0.001 | 0.019 |  |  |
| XA | 0.257 | 0.177 | 0.231 | 0.153 | 0.204 | 0.272 | 0.260 | 0.212 | 0.070 | 0.250 | 0.245 | 0.070 | 0.264 | 0.100 | 0.219 | 0.199 | 0.000 | 0.001 | 0.003 | 0.001 | 0.002 | 0.001 |  |  |
| YA | 0.292 | 0.015 | 0.076 | 0.199 | 0.017 | 0.084 | 0.143 | 0.073 | 0.180 | 0.086 | 0.297 | 0.158 | 0.277 | 0.147 | 0.102 | 0.083 | 0.166 | 0.000 | 0.001 | 0.038 | 0.001 | 0.001 |  |  |
| YC | 0.241 | 0.210 | 0.244 | 0.166 | 0.215 | 0.284 | 0.274 | 0.229 | 0.069 | 0.252 | 0.235 | 0.064 | 0.271 | 0.132 | 0.240 | 0.223 | 0.036 | 0.194 | 0.000 | 0.001 | 0.001 | 0.001 |  |  |
| YK | 0.241 | 0.014 | 0.083 | 0.173 | 0.055 | 0.059 | 0.134 | 0.073 | 0.137 | 0.074 | 0.246 | 0.126 | 0.226 | 0.147 | 0.082 | 0.049 | 0.151 | 0.018 | 0.171 | 0.000 | 0.001 | 0.002 |  |  |
| YT | 0.214 | 0.107 | 0.140 | 0.125 | 0.118 | 0.177 | 0.210 | 0.143 | 0.036 | 0.161 | 0.206 | 0.047 | 0.217 | 0.089 | 0.159 | 0.126 | 0.043 | 0.086 | 0.053 | 0.058 | 0.000 | 0.001 |  |  |
| ZZ | 0.286 | 0.069 | 0.134 | 0.217 | 0.137 | 0.141 | 0.105 | 0.039 | 0.214 | 0.132 | 0.300 | 0.168 | 0.273 | 0.195 | 0.110 | 0.027 | 0.229 | 0.087 | 0.242 | 0.047 | 0.149 | 0.000 |  |  |
| Note: *Fst* Values below diagonal. Probability, P(rand >= data) based on 999 permutations is shown above diagonal. | | | | | | | | | | | | | | | | | | | | | | | |  |
