# Supplementary material for: Genetic Variation May Have Promoted the Successful Colonization of the Invasive Gall Midge, Obolodiplosis robiniae, in China
Source: Front Genet. 2020 Apr 17;11:387. doi: 10.3389/fgene.2020.00387 (PMC7180195; doi:10.3389/fgene.2020.00387)
Supplement: Supplementary file 7 [file Table_4.DOCX]

Table S4. Pairwise Population *Nm* Values Based on *Fst* Values

|  | BJ | CC | CD | DD | DL | DY | GY | HF | NJ | QD | QH | SY | TA | TS | TY | WH | XA | YA | YC | YK | YT | ZZ |
| --- | --- | --- | --- | --- | --- | --- | --- | --- | --- | --- | --- | --- | --- | --- | --- | --- | --- | --- | --- | --- | --- | --- |
| BJ | 0.000 |  |  |  |  |  |  |  |  |  |  |  |  |  |  |  |  |  |  |  |  |  |
| CC | 0.646 | 0.000 |  |  |  |  |  |  |  |  |  |  |  |  |  |  |  |  |  |  |  |  |
| CD | 0.531 | 3.659 | 0.000 |  |  |  |  |  |  |  |  |  |  |  |  |  |  |  |  |  |  |  |
| DD | 1.885 | 1.015 | 0.784 | 0.000 |  |  |  |  |  |  |  |  |  |  |  |  |  |  |  |  |  |  |
| DL | 0.500 | 3.267 | 2.646 | 0.791 | 0.000 |  |  |  |  |  |  |  |  |  |  |  |  |  |  |  |  |  |
| DY | 0.456 | 3.469 | 2.435 | 0.629 | 1.804 | 0.000 |  |  |  |  |  |  |  |  |  |  |  |  |  |  |  |  |
| GY | 0.414 | 1.783 | 1.207 | 0.598 | 1.491 | 1.113 | 0.000 |  |  |  |  |  |  |  |  |  |  |  |  |  |  |  |
| HF | 0.571 | 3.915 | 2.971 | 0.889 | 1.778 | 1.522 | 2.298 | 0.000 |  |  |  |  |  |  |  |  |  |  |  |  |  |  |
| NJ | 0.808 | 1.040 | 0.823 | 1.288 | 0.872 | 0.781 | 0.738 | 0.901 | 0.000 |  |  |  |  |  |  |  |  |  |  |  |  |  |
| QD | 0.462 | 2.324 | 1.334 | 0.626 | 2.967 | 2.234 | 1.734 | 1.401 | 0.905 | 0.000 |  |  |  |  |  |  |  |  |  |  |  |  |
| QH | 5.421 | 0.648 | 0.545 | 3.257 | 0.508 | 0.443 | 0.422 | 0.566 | 0.833 | 0.468 | 0.000 |  |  |  |  |  |  |  |  |  |  |  |
| SY | 0.994 | 1.275 | 0.926 | 1.596 | 0.990 | 0.755 | 0.872 | 1.299 | 6.548 | 0.943 | 0.948 | 0.000 |  |  |  |  |  |  |  |  |  |  |
| TA | 1.303 | 0.773 | 0.596 | 3.401 | 0.536 | 0.553 | 0.443 | 0.596 | 0.869 | 0.512 | 2.151 | 0.910 | 0.000 |  |  |  |  |  |  |  |  |  |
| TS | 1.182 | 1.290 | 1.217 | 4.095 | 1.091 | 0.721 | 0.677 | 1.240 | 1.439 | 0.682 | 1.555 | 1.730 | 1.315 | 0.000 |  |  |  |  |  |  |  |  |
| TY | 0.526 | 1.971 | 1.131 | 0.737 | 1.929 | 1.396 | 2.054 | 1.634 | 0.980 | 5.551 | 0.548 | 1.055 | 0.596 | 0.844 | 0.000 |  |  |  |  |  |  |  |
| WH | 0.611 | 3.986 | 2.778 | 0.882 | 1.850 | 2.268 | 3.373 | 11.050 | 1.086 | 2.053 | 0.575 | 1.366 | 0.629 | 1.110 | 1.981 | 0.000 |  |  |  |  |  |  |
| XA | 0.722 | 1.165 | 0.830 | 1.383 | 0.977 | 0.668 | 0.713 | 0.928 | 3.328 | 0.752 | 0.769 | 3.343 | 0.698 | 2.241 | 0.891 | 1.006 | 0.000 |  |  |  |  |  |
| YA | 0.606 | 16.439 | 3.034 | 1.003 | 14.705 | 2.737 | 1.502 | 3.160 | 1.142 | 2.660 | 0.593 | 1.330 | 0.654 | 1.455 | 2.192 | 2.762 | 1.254 | 0.000 |  |  |  |  |
| YC | 0.786 | 0.943 | 0.774 | 1.260 | 0.911 | 0.629 | 0.664 | 0.841 | 3.389 | 0.743 | 0.815 | 3.676 | 0.673 | 1.649 | 0.793 | 0.873 | 6.792 | 1.041 | 0.000 |  |  |  |
| YK | 0.789 | 17.345 | 2.771 | 1.197 | 4.314 | 3.994 | 1.613 | 3.173 | 1.578 | 3.117 | 0.766 | 1.738 | 0.856 | 1.449 | 2.784 | 4.811 | 1.408 | 13.989 | 1.216 | 0.000 |  |  |
| YT | 0.916 | 2.091 | 1.534 | 1.745 | 1.872 | 1.162 | 0.943 | 1.504 | 6.755 | 1.304 | 0.961 | 5.052 | 0.903 | 2.567 | 1.326 | 1.733 | 5.579 | 2.646 | 4.451 | 4.067 | 0.000 |  |
| ZZ | 0.623 | 3.386 | 1.617 | 0.900 | 1.571 | 1.527 | 2.123 | 6.150 | 0.916 | 1.648 | 0.583 | 1.242 | 0.666 | 1.031 | 2.012 | 9.179 | 0.842 | 2.637 | 0.783 | 5.055 | 1.429 | 0.000 |

*Nm* = 0.25(1 - *Fst*)/*Fs*t.
