# Supplementary material for: Genetic Variation May Have Promoted the Successful Colonization of the Invasive Gall Midge, Obolodiplosis robiniae, in China
Source: Front Genet. 2020 Apr 17;11:387. doi: 10.3389/fgene.2020.00387 (PMC7180195; doi:10.3389/fgene.2020.00387)
Supplement: Supplementary file 8 [file Table_5.DOC]

| Table S5. Genetic identity and genetic distance for *O. robiniae* samples based on Nei (1978) | | | | | | | | | | | | | | | | | | | | | | |
| --- | --- | --- | --- | --- | --- | --- | --- | --- | --- | --- | --- | --- | --- | --- | --- | --- | --- | --- | --- | --- | --- | --- |
| **Pop** | **BJ** | **CC** | **CD** | **DD** | **DL** | **DY** | **GY** | **HF** | **NJ** | **QD** | **QH** | **SY** | **TA** | **TS** | **TY** | **WH** | **XA** | **YA** | **YC** | **YK** | **YT** | **ZZ** |
| BJ | **** | 0.572 | 0.493 | 0.833 | 0.499 | 0.526 | 0.428 | 0.489 | 0.655 | 0.492 | 0.939 | 0.679 | 0.803 | 0.726 | 0.5 | 0.534 | 0.613 | 0.546 | 0.646 | 0.631 | 0.669 | 0.554 |
| CC | 0.558 | **** | 0.901 | 0.663 | 0.903 | 0.924 | 0.841 | 0.899 | 0.698 | 0.876 | 0.548 | 0.713 | 0.648 | 0.715 | 0.839 | 0.904 | 0.726 | 0.962 | 0.669 | 0.961 | 0.825 | 0.893 |
| CD | 0.705 | 0.104 | **** | 0.579 | 0.885 | 0.896 | 0.777 | 0.876 | 0.631 | 0.799 | 0.477 | 0.622 | 0.557 | 0.706 | 0.738 | 0.873 | 0.631 | 0.886 | 0.611 | 0.87 | 0.775 | 0.799 |
| DD | 0.182 | 0.41 | 0.545 | **** | 0.614 | 0.587 | 0.521 | 0.593 | 0.732 | 0.546 | 0.891 | 0.744 | 0.905 | 0.885 | 0.564 | 0.602 | 0.747 | 0.659 | 0.725 | 0.696 | 0.776 | 0.614 |
| DL | 0.695 | 0.101 | 0.121 | 0.487 | **** | 0.873 | 0.831 | 0.825 | 0.676 | 0.909 | 0.477 | 0.674 | 0.542 | 0.701 | 0.853 | 0.836 | 0.708 | 0.966 | 0.691 | 0.921 | 0.83 | 0.812 |
| DY | 0.641 | 0.079 | 0.109 | 0.531 | 0.134 | **** | 0.807 | 0.831 | 0.692 | 0.898 | 0.485 | 0.639 | 0.615 | 0.621 | 0.831 | 0.888 | 0.638 | 0.907 | 0.621 | 0.932 | 0.775 | 0.846 |
| GY | 0.847 | 0.172 | 0.251 | 0.652 | 0.185 | 0.214 | **** | 0.868 | 0.639 | 0.858 | 0.407 | 0.651 | 0.477 | 0.551 | 0.867 | 0.909 | 0.624 | 0.815 | 0.603 | 0.82 | 0.693 | 0.867 |
| HF | 0.714 | 0.106 | 0.131 | 0.521 | 0.191 | 0.185 | 0.141 | **** | 0.633 | 0.795 | 0.453 | 0.699 | 0.522 | 0.684 | 0.799 | 0.951 | 0.64 | 0.881 | 0.61 | 0.874 | 0.751 | 0.927 |
| NJ | 0.421 | 0.359 | 0.459 | 0.312 | 0.39 | 0.368 | 0.446 | 0.456 | **** | 0.707 | 0.646 | 0.929 | 0.686 | 0.743 | 0.695 | 0.701 | 0.892 | 0.721 | 0.894 | 0.784 | 0.932 | 0.653 |
| QD | 0.708 | 0.132 | 0.224 | 0.604 | 0.094 | 0.106 | 0.152 | 0.228 | 0.346 | **** | 0.47 | 0.681 | 0.549 | 0.56 | 0.943 | 0.86 | 0.647 | 0.89 | 0.648 | 0.901 | 0.777 | 0.837 |
| QH | 0.062 | 0.601 | 0.739 | 0.115 | 0.738 | 0.722 | 0.898 | 0.791 | 0.436 | 0.754 | **** | 0.641 | 0.869 | 0.774 | 0.49 | 0.476 | 0.614 | 0.508 | 0.638 | 0.597 | 0.664 | 0.496 |
| SY | 0.387 | 0.337 | 0.474 | 0.294 | 0.394 | 0.447 | 0.428 | 0.357 | 0.073 | 0.383 | 0.443 | **** | 0.659 | 0.745 | 0.675 | 0.722 | 0.879 | 0.724 | 0.889 | 0.772 | 0.904 | 0.696 |
| TA | 0.219 | 0.433 | 0.584 | 0.098 | 0.611 | 0.484 | 0.739 | 0.649 | 0.375 | 0.598 | 0.139 | 0.416 | **** | 0.761 | 0.565 | 0.559 | 0.61 | 0.586 | 0.599 | 0.666 | 0.673 | 0.598 |
| TS | 0.319 | 0.335 | 0.347 | 0.121 | 0.354 | 0.476 | 0.594 | 0.379 | 0.296 | 0.579 | 0.255 | 0.293 | 0.273 | **** | 0.595 | 0.66 | 0.827 | 0.745 | 0.773 | 0.73 | 0.83 | 0.633 |
| TY | 0.693 | 0.175 | 0.303 | 0.572 | 0.158 | 0.184 | 0.142 | 0.224 | 0.363 | 0.058 | 0.712 | 0.393 | 0.569 | 0.518 | **** | 0.836 | 0.663 | 0.853 | 0.629 | 0.876 | 0.752 | 0.848 |
| WH | 0.625 | 0.101 | 0.134 | 0.506 | 0.178 | 0.118 | 0.095 | 0.051 | 0.355 | 0.151 | 0.741 | 0.325 | 0.581 | 0.415 | 0.178 | **** | 0.676 | 0.871 | 0.635 | 0.913 | 0.787 | 0.952 |
| XA | 0.488 | 0.32 | 0.459 | 0.291 | 0.345 | 0.448 | 0.47 | 0.445 | 0.113 | 0.434 | 0.487 | 0.128 | 0.493 | 0.189 | 0.41 | 0.391 | **** | 0.742 | 0.937 | 0.759 | 0.923 | 0.616 |
| YA | 0.605 | 0.038 | 0.121 | 0.416 | 0.033 | 0.097 | 0.203 | 0.125 | 0.326 | 0.116 | 0.675 | 0.322 | 0.533 | 0.294 | 0.158 | 0.137 | 0.297 | **** | 0.697 | 0.956 | 0.857 | 0.865 |
| YC | 0.435 | 0.401 | 0.493 | 0.32 | 0.369 | 0.477 | 0.505 | 0.493 | 0.111 | 0.433 | 0.448 | 0.117 | 0.512 | 0.256 | 0.463 | 0.454 | 0.064 | 0.359 | **** | 0.727 | 0.907 | 0.594 |
| YK | 0.461 | 0.039 | 0.138 | 0.361 | 0.082 | 0.069 | 0.198 | 0.134 | 0.243 | 0.103 | 0.515 | 0.257 | 0.405 | 0.314 | 0.131 | 0.091 | 0.275 | 0.044 | 0.318 | **** | 0.895 | 0.922 |
| YT | 0.401 | 0.191 | 0.254 | 0.252 | 0.185 | 0.254 | 0.366 | 0.286 | 0.069 | 0.251 | 0.409 | 0.1 | 0.396 | 0.186 | 0.284 | 0.238 | 0.079 | 0.154 | 0.096 | 0.111 | **** | 0.747 |
| ZZ | 0.589 | 0.113 | 0.223 | 0.486 | 0.207 | 0.166 | 0.142 | 0.074 | 0.424 | 0.177 | 0.7 | 0.361 | 0.513 | 0.455 | 0.164 | 0.048 | 0.483 | 0.144 | 0.52 | 0.081 | 0.291 | **** |
| Genetic distance values are below diagonal and Genetic Identity values are above diagonal. | | | | | | | | | | | | | | | | | | | | | | |
